# Dental hygiene and dental students’ motivations for future work: a cross-sectional study of first-year students at a dental hygiene school and a dental school in Japan

**DOI:** 10.1186/s12909-023-04864-3

**Published:** 2023-11-16

**Authors:** Yasuyuki Takahashi, Asami Iguchi, Shiho Motoi, Mio Susuga, Yuh Hasegawa

**Affiliations:** 1 Dental Anesthesia and General Health Management, The Nippon Dental University Niigata Hospital, 1-8 Hamaura-cho, Chuo-ku, Niigata, 951-8580 Japan; 2 Department of Dental Anesthesiology, School of Life Dentistry at Niigata, The Nippon Dental University, Niigata, Japan; 3 Department of Dental Hygiene, The Nippon Dental University College at Niigata, Niigata, Japan

**Keywords:** Dental hygiene students, Dental students, Career choice, Career perception, Female students

## Abstract

**Background:**

It is important to understand the career motivations and perceptions of students who intend to become dental health professionals. Both dental and dental hygiene students may have different opinions about the profession and future work prospects. To our knowledge, no study has compared the career motivations and career perceptions of Japanese dental and dental hygiene students after admission to dental or dental hygiene school. This cross-sectional study examined the motivations of dental and dental hygiene students for their future career perceptions.

**Methods:**

First-year students of dental and dental hygiene schools in the academic years 2021 and 2022 participated in the study. Group 1 comprised 104 female dental hygiene students, Group 2−1 comprised 55 female dental students, and Group 2–2 comprised 61 male dental students. A self-administered questionnaire survey was conducted on opinions of the work and prospects of future work with four-point Likert scales ranging from “strongly agree” to “strongly disagree,” according to the items.

**Results:**

Dental hygiene students consider that they would like to work as professionals and would also like to earn efficiently. Specifically, female dental students prioritized contributing to society by working long-term in a fulfilling environment rather than earning an income, whereas male dental students would like to work as a professional while also effectively earning income.

**Conclusions:**

Small but significant differences were found in opinions of the dental hygienist/dentist profession in terms of attractiveness and credibility. Small but significant differences were found for income and working hours when considering future job prospects. The results of this study revealed differences in the first year students between dental and dental hygiene school in their attitudes toward career motivation and prospects for future work.

## Background

In Japan, most of the students enter dental and dental hygiene schools immediately after graduating high school. Dental hygiene education in Japan includes three types of programs: the four-year baccalaureate degree, three-year associate degree, and three-year diploma title. Most dental hygiene schools in Japan have a three-year program. Dental hygiene students attend lectures on general sciences and basic clinical practice, clinical subjects and clinical practice, and clinical practice and the Japanese national board examination for dental hygienist lectures in the first, second, and third years respectively. Dental schools in Japan follow a six-year course program. An example of a dental school curriculum adopted in one private school is as follows. The first year includes general sciences, with preclinical training in the second through fourth years, clinical internship at the university clinic in the fifth year, and classroom lectures to prepare for the Japanese national board examination for dentists in the sixth year. For frequent tasks, in Japan, dental hygienists are required by law to treat patients under the supervision of dentists [1]. The three main responsibilities of dental hygienists have been defined as preventive treatment of dental caries and periodontal disease, assisting the dentist during dental treatment, and oral health education [1, 2]. In Japan, students of dental and dental hygiene schools must pay tuition, as these schools are not free.

Previous reports have demonstrated that female healthcare workers may experience career breaks due to pregnancy, childbirth, and child-rearing [3–5]. In recent years, the percentage of female students in dental and dental hygiene schools has been increasing worldwide [4, 6, 7]; specifically, in Japan, most dental hygiene students are female [3]. Many female students consider their career path based on the possibility of marriage and child-rearing in the future [8]. Subsequently, it is important to understand the career motivations and related perceptions of the students who intend to become dental health professionals. The reports from U.S. expressed that students who intend to become health care professionals, including dental hygienists and dentists, already have a clear idea of their future work when they enter a training school [9, 10]. It is likely that there are differences between countries in terms of the structure of dentistry, possibilities for women to work, and incomes, which may influence one’s motivation to enter dental or dental hygiene schools. Opinions toward profession and prospects for future work may differ between female dental and dental hygiene students. To our knowledge, no study has compared Japanese dental and dental hygiene students’ career motivations and related perceptions after admission to dental or dental hygiene schools. Therefore, the present study conducted a questionnaire survey in the first term of the first year to determine career motivation after entering schools. The null hypothesis was that there would be no difference between female dental and dental hygiene students’ career motivations and perceptions for future work.

## Methods

This cross-sectional study aimed to investigate the attitudes of dental and dental hygiene students regarding their future career choices.

### Study group

**Fig. 1:**
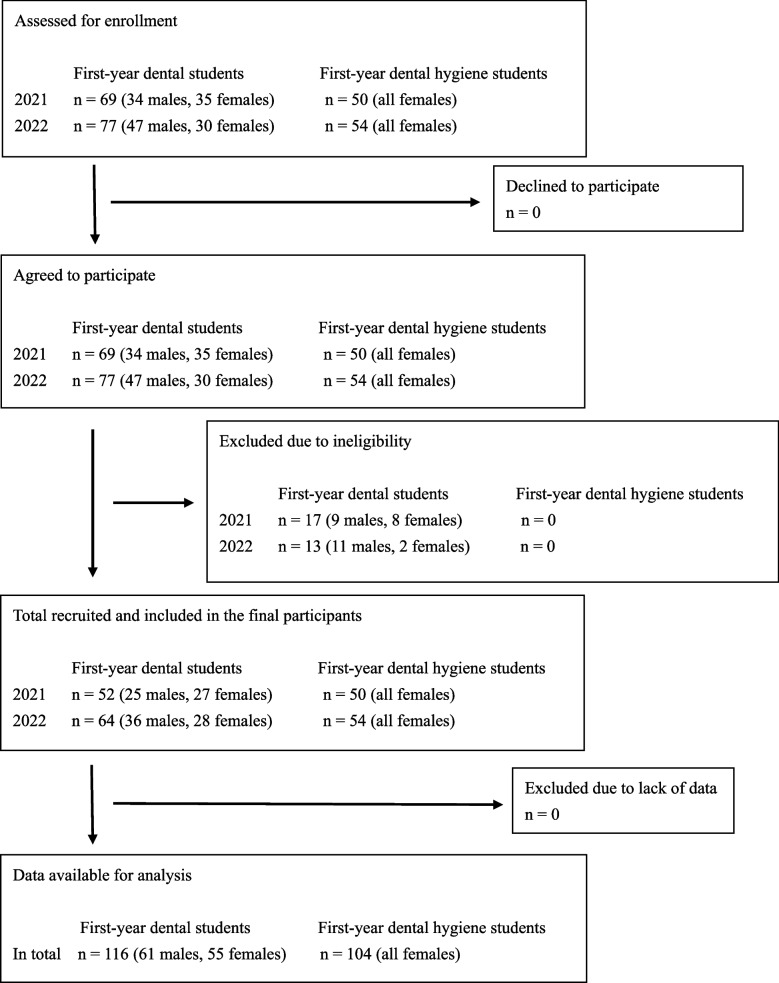
The sampling process is described in the flow chart

The Nippon Dental University College at Niigata has adopted a three-year educational program, and The Nippon Dental University, School of Life Dentistry at Niigata has adopted a six-year educational program. First-year students of dental (The Nippon Dental University, School of Life Dentistry at Niigata) and dental hygiene schools (The Nippon Dental University College at Niigata) in the academic years 2021 and 2022 participated in the study. In 2021 and 2022, the first-year dental hygiene students were all female. The sampling process described in the STROBE flowchart is illustrated in Fig. 1. A total of 220 students (104 female dental hygiene students, 61 male dental, and 55 female dental students; all Japanese) were divided into three groups. Group 1 comprised 104 female dental hygiene students, Group 2−1 comprised 55 female dental students, and Group 2–2 comprised 61 male dental students. All participants signed the agreement on a consent form and written informed consent was obtained from them.

### Questionnaire survey

A self-administered paper-and-pencil style questionnaire survey was conducted on the impressions of the work and prospects for future work. The items on the questionnaire survey were “opinion of dental hygienist (for dental hygiene students)/dentist (for dental students) profession” and “prospects for future work.” Participants were asked to rank their thoughts for each question on a four-point Likert scale ranging from 1 (strongly disagree) to 4 (strongly agree) according to the items: strongly disagree; disagree; agree; and strongly agree. The questions consisted of seven items, each labeled by “1. Opinions” and “2. Prospects” as follows: (1) It is a worthwhile profession; (2) I can make a social contribution through the profession; (3) It is a rigorous and arduous profession; (4) The profession entails responsibility; (5) It is a delightful profession; (6) The profession enhances my ability; (7) The profession strengthens my credibility; (8) I want a rewarding job; (9) I want to help others; (10) I want to earn as much money as possible; 11) I want to work with minimal workload and stress; 12) I want to work as little as possible; 13) I want to be able to continue working for a long period; 14) I want to live without working, if possible; and personal attributes (year-grade, sex, and faculty). The survey utilized a questionnaire instrument originally developed by our research team and previously developed and successfully used with dental hygiene students at the same institute [11]. The survey was conducted in September 2021 and 2022 for the dental hygiene students and in July 2021 and 2022 for the dental students. There was no relationship between the researcher, who carried out the questionnaire, and the student participants (e.g., teacher-student). The questionnaire was administered after school hours.

### Statistics and data analysis

The sample size was calculated based on a power analysis using G* Power Software version 3.1.9.2 (Heinrich Heine University, Dusseldorf, Germany) to determine the power of statistical analysis. The power analysis for the Mann-Whitney U test was calculated with an alpha error probability of 0.05 and a power of 80%, indicating that a total of 134 samples (67 in each group) were required for the test. The power analysis results demonstrated that the sample size (104 female dental hygiene students, 116 dental students; 220 in total) of the present study was sufficient for statistical comparisons. The Mann-Whitney U test was used to clarify the differences in the mean values between the dental and dental hygiene student groups for the impression and prospection scores. The power analysis for the Kruskal-Wallis test was calculated with an alpha error probability of 0.05 and a power of 80%, indicating that a total of 159 samples (53 in each group) were required for the test. The power analysis results demonstrated that the sample size (104 female dental hygiene students, 61 male dental, and 55 female dental students; 220 in total) of the present study was sufficient for statistical comparisons. Regarding the participants’ evaluation on a scale of values ranging from 1 (strongly disagree) to 4 (strongly agree) for each question, the mean and standard deviation were calculated respectively. The Kruskal-Wallis test was used to analyze the scores in the mean values among the three student groups for the impression and prospection scores. If a significant difference was observed, a Holm multiple comparison test was performed to identify significant differences between the groups. The significance level (alpha) was set at 0.05 for all statistical analyses, and all statistical analyses were performed using the IBM SPSS Statistics Version 28.0.0. software.

## Results

### Summary of respondents

Through the academic years 2021 and 2022, 104 dental hygiene students (all females) and 146 dental students (81 males and 65 females) were assessed for enrollment in the present study. The number of students excluded as ineligible because they “did not complete questionnaires by the deadline” or “had an experience of repeating a year” were 0 in the dental hygiene student group and 17 in the dental student group (9 males and 8 females) in 2021, and 0 in the dental hygiene student group and 13 in the dental student group (11 males and 2 females) in 2022, respectively. None of the dental and dental hygiene students were excluded owing to a lack of data. Finally, data collected from 104 dental hygiene students (all females) and 116 dental students (61 males and 55 females) were available for analysis. The overall participation rate was 88.0% (100% of the dental hygiene students and 79.5% of the dental students, including 75.3% for males and 84.6% for females).

### Intergroup comparison

Table 1 illustrates the comparison results between the dental and dental hygiene students in terms of their opinions of the work of the dental hygienists and dentists. The results showed no significant differences between dental and dental hygiene students on the questions 1 through 4. However, questions 5 “It is a delightful profession,” 6 “The profession enhances my ability,” and 7 “The profession strengthens my credibility” showed small but significant differences between dental and dental hygiene students. Table 2 illustrates the comparison results among the dental hygiene female student group and the female and male dental student group concerning their opinions of the work of the dental hygienists and dentists. This study’s findings showed no significant differences among female dental hygiene students, female dental students, and male dental students on the questions 1 through 4. However, questions 5 “It is a delightful profession” and 6 “The work enhances my ability” showed small but significant differences between female dental and dental hygiene students; whereas for question 7 “The profession strengthens my credibility” in the present study, we observed small but significant discrepancies between female dental and dental hygiene students, and between female dental hygiene and male dental students, but not between female and male dental students.

**Table 1:** Opinion of dental hygienist/dentist work (1 = strongly disagree, 2 = disagree, 3 = agree, and 4 = strongly agree)

	Group 1 (Female dental hygiene students)		Group 2 (Female and male dental students)		
Questions	Mean	SD	Mean	SD	Mann-Whitney U test
1 It is a worthwhile profession	3.7	0.5	3.8	0.4	*NS*
2 I can make a social contribution through the profession	3.8	0.5	3.9	0.4	NS
3 It is a rigorous and arduous profession	3.7	0.6	3.8	0.4	*NS*
4 The profession entails responsibility	3.9	0.4	4.0	0.2	NS
5 It is a delightful profession	2.9	0.7	3.2	0.8	*p* < 0.01
6 The profession enhances my ability	3.5	0.5	3.8	0.5	*p* < 0.01
7 The profession strengthens my credibility	3.5	0.6	3.7	0.5	*p* < 0.01

*SD* Standard deviation, *NS* Not significant

**Table 2:** Opinion of dental hygienist/dentist work (1 = strongly disagree, 2 = disagree, 3 = agree, and 4 = strongly agree)

	Group 1 (Female dental hygiene students)		Group 2-1 (Female dental students)		Group 2-2 (Male dental students)			Comparison among groups (Holm)		
Questions	Mean	SD	Mean	SD	Mean	SD	Kruskal-Wallis test	1 vs. 2-1	1 vs. 2-2	2-1 vs. 2-2
1 It is a worthwhile profession	3.7	0.5	3.9	0.3	3.8	0.4	NS			
2 I can make a social contribution through the profession	3.8	0.5	3.9	0.4	3.9	0.4	NS			
3 It is a rigorous and arduous profession	3.7	0.6	3.8	0.5	3.8	0.4	NS			
4 The profession entails responsibility	3.9	0.4	4.0	0.0	4.0	0.2	NS			
5 It is a delightful profession	2.9	0.7	3.2	0.8	3.2	0.8	*p* < 0.01	*p* < 0.01	NS	NS
6 The profession enhances my ability	3.6	0.5	3.8	0.4	3.7	0.6	*p* < 0.01	*p* < 0.05	NS	NS
7 The profession strengthens my credibility	3.5	0.6	3.8	0.5	3.7	0.51	*p* < 0.01	*p* < 0.05	*p* < 0.05	NS

*SD* Standard deviation, *NS* Not significant

Table 3 illustrates the results of the comparisons between the dental and dental hygiene students in their perspectives for future work. There were no significant differences on questions 8, 9, 13, and 14. Question 10 “I want to earn as much money as possible” showed small but significant differences between female dental and dental hygiene students. Questions 11 “I want to work with minimal workload and stress” and 12 “I want to work as little as possible” showed small but significant differences. Table 4 illustrates the results of the comparisons among the female dental hygiene student group and the female and male dental student group in their perspectives for future work. There were no significant differences among female dental hygiene, female dental, and male dental students on questions 8 and 9. Question 10 “I want to earn as much money as possible” showed small but significant differences between female dental and dental hygiene students. Questions 11 “I want to work with minimal workload and stress” and 12 “I want to work as little as possible” showed small but significant differences between female dental and dental hygiene students and between female dental hygiene and male dental students; whereas for question 13 “I want to be able to continue working for a long period,” we observed small but significant differences between female and male dental students. Question 14 “I want to live without working, if possible” showed small but significant differences between female dental and dental hygiene students, and between female dental and male dental students, but not between female dental hygiene and male dental students.

**Table 3:** Prospects for future work (1 = strongly disagree, 2 = disagree, 3 = agree, and 4 = strongly agree)

	Group 1		Group 2		
	(Female dental hygiene students)		(Female and male dental students)		
Questions	Mean	SD	Mean	SD	Mann-Whitney U test
8 I want a rewarding job	3.8	0.4	3.9	0.3	NS
9 I want to help others	3.8	0.5	3.9	0.4	NS
10 I want to earn as much money as possible	3.7	0.5	3.4	0.7	*p* < 0.01
11 I want to work with minimal workload and stress	3.3	0.7	2.8	0.9	*p* < 0.01
12 I want to work as little as possible	3.1	0.8	2.7	0.9	*p* < 0.01
13 I want to be able to continue working for a long period	3.8	0.4	3.7	0.6	NS
14 I want to live without working, if possible	2.3	1.2	2.0	1.2	NS

*SD* Standard deviation, *NS* Not significant

**Table 4:** Prospects for future work (1 = strongly disagree, 2 = disagree, 3 = agree, and 4 = strongly agree)

	Group 1		Group 2−1		Group 2–2					
	(Female dental hygiene students)		(Female dental students)		(Male dental students)			Comparison among groups (Holm)		
Questions	Mean	SD	Mean	SD	Mean	SD	Kruskal-Wallis test	1 vs. 2−1	1 vs. 2–2	2−1 vs. 2–2
8 I want a rewarding job	3.8	0.4	3.9	0.3	3.9	0.3	NS			
9 I want to help others	3.8	0.5	3.9	0.4	3.8	0.4	NS			
10 I want to earn as much money as possible	3.7	0.5	3.3	0.7	3.5	0.7	*p* < 0.001	*p* < 0.01	NS	NS
11 I want to work with minimal workload and stress	3.3	0.7	2.6	0.8	3.1	0.9	*p* < 0.001	*p* < 0.001	NS	*p* < 0.01
12 I want to work as little as possible	3.1	0.8	2.5	0.8	3.0	1.0	*p* < 0.001	*p* < 0.001	NS	*p* < 0.01
13 I want to be able to continue working for a long period	3.8	0.4	3.5	0.7	3.9	0.3	*p* < 0.01	NS	NS	*p* < 0.05
14 I want to live without working, if possible	2.3	1.2	1.7	1.0	2.3	13.5	*p* < 0.01	*p* < 0.01	NS	*p* < 0.05

*SD* Standard deviation, *NS* Not significant

## Discussion

The female dental students were more likely, than the female dental hygiene students, to perceive their chosen profession as delightful and believe that their profession would enhance their abilities. McGregor et al. [12] reported that, because few dental hygiene programs in the United States are affiliated with dental schools, an isolated training environment for dentists may perpetuate the traditional perception that their role is mono-professional. The present study’s results suggest some kind of disagreement between dental and dental hygiene students about the credibility of their profession. Female dental hygiene students may enter their profession with the perception that they are fundamentally dependent on other healthcare team members. Dental education may inadvertently focus on dentists as leaders of the oral healthcare team [12, 13], and this may be because a dental hygienist in Japan must work under instruction, while dentists practice independently. Based on their research in the United States, Shaikh and Inglehart [14] found that dental students were more likely than dental hygiene students to choose their career because they wanted to help others and because of the practical aspects of dentistry, human interaction, lifestyle, science-related, and business-related considerations.

In a previous study in Japan [15], a professional license was the most common reason for choosing dental hygiene as a career and the most appealing aspect of becoming a dental hygienist. Professional licenses have long been highly valued in Japanese society and can be a deciding factor in pursuing further education. Thus, we consider that the dental hygiene students in this study are willing to work continuously for a long period utilizing their professional licenses. Dental students with family members in healthcare professions were likelier to be motivated by being inspired by human interaction aspects of the profession than students who did not have such professionals in their family [7, 14]. However, Rabeeah et al. [16] reported that merely having a dental hygienist or dentist in the family did not correlate with higher job satisfaction. It is of interest to note that the dental hygiene students in the present study were willing to continue working for a long period, and it is necessary to examine the influence of their family background on their career perceptions in further research. Faculty members need to understand the characteristics of these dental hygiene students because their attitudes toward national licenses and career perceptions are thought to differ from those of dental students.

In the present study, the willingness of female dental students to continue working for a lifetime was the lowest, but not significantly so and they were significantly unwilling to live without working. Female dental students are considered positive about working in the future and want to work with consistence [7, 17, 18]. A woman’s life is characterized by turning points, such as marriage, childbirth, and child-rearing. Dental services in Japan are primarily provided through private dental practices. The previous reports from Japan suggest that young dentists working in hospitals have long working hours and inadequate environments to support childcare [19] and that the working hours of female dentists employed in private dental practices are shorter than those who are owners of such practices [20]. Furthermore, Oshima et al. [21] reported that female dentists were less likely than male dentists to become the owners of dental practices and, once resuming work, female dentists were more likely to work part-time in dental practices. Based on these reports and the results of this study, it is considered that female dentists attempt to balance work and domestic responsibilities once resuming work to continue working as long as possible. In Japan, domestic responsibilities, especially parental duties, are shouldered more by women than men [22]. Furthermore, traditionally, it has been a custom for men to earn in Japan. Male dental students are likely to prioritize efficiency in their future careers, have more working hours across their careers, and take fewer career breaks than female dentists [23]. In the present study, the job perceptions of female dental hygiene students were similar to those of male dental students. It was previously reported that female dental students tended to be more emotionally expressive and sensitive than male dental students [24, 25]. To apply the differences in career awareness of female dental and dental hygiene students identified in this study, we believe that career interruption and subsequent reemployment should be adopted as a career education topic for dental and dental hygiene students. Furthermore, it would be important for male students to be aware of the career interruption and the subsequent reemployment of women due to marriage, childbirth, and child-rearing, so that both genders are on the same level. Regarding the differences between female dental and dental hygiene students, it is possible that the grades required to enter the dental and dental hygiene programs, the opportunity to find a job after graduation, and the length of said programs may also influence applicants’ and students’ motivation. Further research is needed to determine the influences of these factors on the motivation of both applicants and students.

## Limitations

This study has a few limitations. First, data were collected at one dental hygiene college and one dental university only, limiting the generalizability of the findings. Second, this study had a cross-sectional design, while two years of data were employed for the analysis. Although the results of the statistical analyses showed significant differences, all mean differences appeared to be small and did not exceed 0.3. Continuous data collection and a longitudinal study design are required to clarify further career perceptions of dental and dental hygiene students.

## Conclusions

In conclusion, based on the study’s findings, the null hypothesis was rejected. Significant differences were found in opinions of the dental hygienist/dentist profession in terms of attractiveness and credibility. Regarding the prospects for future work, significant differences were found for income and working hours. Moreover, the findings revealed differences between dental and dental hygiene first-year students in their attitudes toward career motivation and prospects for future work.

## Data Availability

The datasets used and/or analyzed during the current study are available from the corresponding author upon reasonable request.
